# Using Artificial Intelligence-Enhanced Sensing and Wearable Technology in Sports Medicine and Performance Optimisation

**DOI:** 10.3390/s22186920

**Published:** 2022-09-13

**Authors:** Swathikan Chidambaram, Yathukulan Maheswaran, Kian Patel, Viknesh Sounderajah, Daniel A. Hashimoto, Kenneth Patrick Seastedt, Alison H. McGregor, Sheraz R. Markar, Ara Darzi

**Affiliations:** 1 Department of Surgery & Cancer, Imperial College London, St. Mary’s Hospital, London W2 1NY, UK; 2 Institute of Global Health Innovation, Imperial College London, South Kensington Campus, London SW7 2AZ, UK; 3 Department of Surgery, University of Pennsylvania Perelman School of Medicine, Philadelphia, PA 19104, USA; 4 Department of Surgery, Beth Israel Deaconess Medical Center, Boston, MA 02215, USA; 5 Musculoskeletal Laboratory, Division of Surgery, Department of Surgery and Cancer, Faculty of Medicine, Imperial College London, White City Campus, London W12 OBZ, UK; 6 Department of Molecular Medicine and Surgery, Karolinska Institutet, 171 76 Stockholm, Sweden; 7 Nuffield Department of Surgical Sciences, Department of Surgery, Churchill Hospital, Old Road, Headington, Oxford OX3 7LE, UK

**Keywords:** artificial intelligence, sports medicine, sensors, wearables

## Abstract

Wearable technologies are small electronic and mobile devices with wireless communication capabilities that can be worn on the body as a part of devices, accessories or clothes. Sensors incorporated within wearable devices enable the collection of a broad spectrum of data that can be processed and analysed by artificial intelligence (AI) systems. In this narrative review, we performed a literature search of the MEDLINE, Embase and Scopus databases. We included any original studies that used sensors to collect data for a sporting event and subsequently used an AI-based system to process the data with diagnostic, treatment or monitoring intents. The included studies show the use of AI in various sports including basketball, baseball and motor racing to improve athletic performance. We classified the studies according to the stage of an event, including pre-event training to guide performance and predict the possibility of injuries; during events to optimise performance and inform strategies; and in diagnosing injuries after an event. Based on the included studies, AI techniques to process data from sensors can detect patterns in physiological variables as well as positional and kinematic data to inform how athletes can improve their performance. Although AI has promising applications in sports medicine, there are several challenges that can hinder their adoption. We have also identified avenues for future work that can provide solutions to overcome these challenges.

## 1. Introduction

Wearable technology refers to small electronic and mobile devices with wireless communication capabilities that can be worn on the body as a part of gadgets, accessories or clothes [1]. They are mechanical devices or intelligent mechatronic systems that often incorporate sensors. The size of the global wearable technology market was valued at USD 40.65 billion in 2020 and is expected to rise further by 13.8% before 2028 [2]. This is primarily due to increased availability, reduced costs and greater potential for their incorporation into new domains, such as sports medicine. In healthcare, they provide a promising avenue for the diagnosis, monitoring and management of medical conditions. They are useful in collating data about physiological parameters by measuring blood pressure, heart rate, body temperature, electrocardiogram (ECG), electroencephalogram (EEG), sweat analysis and movement data such as displacement, velocity and acceleration. Improvements in design enable the capture of these data in a meaningful way for application in healthcare. For example, the Oura ring has been validated using actigraphy methods as an accurate measure to monitor sleep [3,4]. Apple Health is using the Apple Watch to collect data as a part of two separate research studies, including the Apple Heart and Movement Study as well as the Apple Hearing Study, to better inform the screening and risk assessment of conditions [5]. Overall, there is an increasing trend in the use of wearable devices in healthcare.

The intersection of large volumes of data and healthcare applications calls for the use of artificial intelligence (AI) technology to improve how the data are handled, processed and used. Russell and Norvig define artificial intelligence as the designing and building of intelligent agents that receive precepts from the environment and take actions that affect that environment [6]. In sports medicine, this can be better understood as technology that emulates human tasks, often using machine learning as the method to learn from data how to emulate these task. Machine learning (ML) is a subset of AI methodology that uses data to classify, predict or gain useful inference without explicit instructions [7]. By inputting data into ML systems, they can be trained to carry out tasks in supervised, unsupervised or semi-supervised environments. ML-based algorithms have been used elsewhere in healthcare research for both the diagnosis and monitoring of diseases [8]. Extending their application to wearable technology and the data they collect can dramatically change the way patient data are used. Previous work has demonstrated the utility of AI in chronic conditions, such as cardiovascular conditions, cancers and neurological diseases. Sports medicine can be an impactful domain for the integration of AI due to the increasing prevalence of wearable technology. Of the various fields for their application in healthcare, the use of sensors is most widespread in sports medicine, specifically in injury prevention, risk management, performance optimisation and obtaining sporting advantages. However, there is a paucity of work summarising the combined use of AI and wearable technology use in sports medicine. Narrative reviews are useful to attain a broad perspective on a topic using the most pivotal studies while retaining the scientific accuracy. In this narrative review, we explore how artificial intelligence has been applied into wearable technologies within healthcare.

## 2. Methods

### 2.1. Literature Search

The following databases were searched: MEDLINE (1946 until the first week of March 2022) via OvidSP; MEDLINE in-process and other non-indexed citations (latest issue) via OvidSP; Ovid EMBASE (1974 to latest issue); (d) Scopus (1996 till present). The last search was performed on 13 March 2022. Three separate searches were performed to include all articles that evaluated the use of artificial intelligence and machine learning alongside wearable devices for healthcare applications. Search terms used several strings, which were linked by standard modifiers in the following order: wearable devices OR wearable technology OR wearables OR healthcare sensors OR sports sensors OR smart wears OR industrial wearable OR sports wearable OR heart rate monitors OR accelerometers. The second search included the following terms: artificial intelligence OR AI OR data mining OR deep learning OR machine learning OR ML. The third string included: sports OR athlete OR sports medicine OR sports performance. The strings were then combined using the AND modifier.

### 2.2. Selection and Quality Assessment of Studies

Articles were screened for eligibility by SC and YM, and, where required, the third co-author (VS) was consulted. Studies were included if they had investigated the use of AI in wearable devices for healthcare applications, specifically in relation to sports medicine. Studies with diagnostic, prognostic and monitoring intents were included. We classified these studies into the phase of sporting event as before, during and after the event. Studies were excluded if they did not evaluate AI; did not use a wearable device or sensor to collect data; were not carried out in the context of sports medicine; had incomplete data on outcome measures; were not in the English language; or had incompatible designs including letters, comments and reviews. Studies were assessed for robustness of methodology using the quality assessment tool for diagnostic accuracy studies 2 (QUADAS-2). The QUADAS-2 comprises four domains covering patient selection, index test, reference standard and flow of patients through the study and timing of the index test(s) and reference standard. Each domain is evaluated in terms of the risk of bias, and the first three are also assessed for any concerns regarding applicability. In doing so, it highlights aspects of the study design that may be exposed to bias.

**Figure 1:**
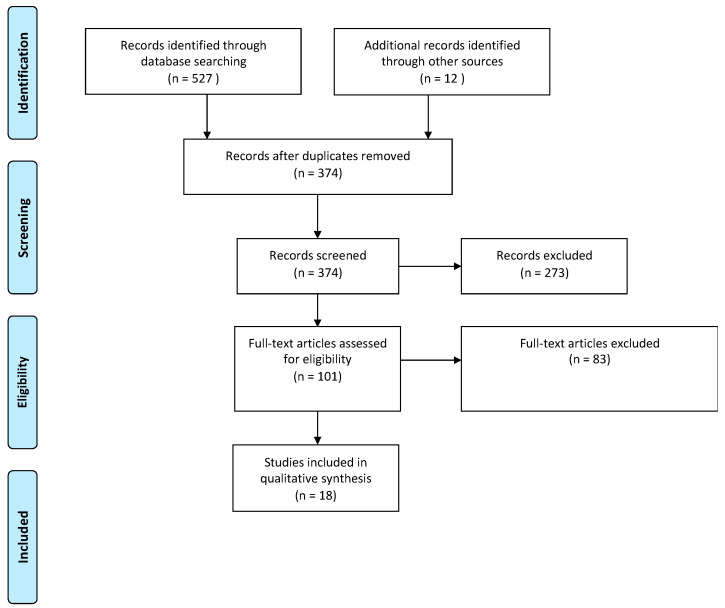
PRISMA diagram of included studies.

**Figure 2:**
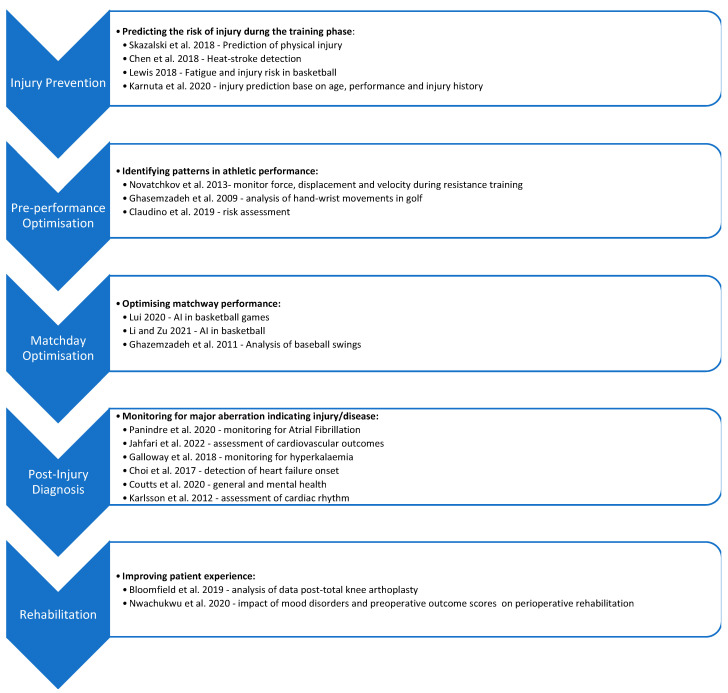
Classification of studies reporting sensors’ use in the different phases of sports [9,10,11,12,13,14,15,16,17,18,19,20,21,22,23,24,25,26].

The database search yielded a total of 527 studies. After duplications were removed, titles and abstracts of the remaining 374 studies were assessed for eligibility, and 273 studies were removed. A further 80 studies were excluded after full-text review due to incompatible outcome measures or study design (Figure 1). Of the remaining studies that discussed the use of artificial intelligence in sensors for sports medicine, we classified them according to each phase of an athlete’s journey, which typically consists of pre-event training, in-event performance, injuries during events or training phases, and follow-up if athletes sustain any injuries. Based on this journey, we categorised studies based on their ability to predict the risk of injury during training or events; optimisation of performance; diagnosis of injuries; and management of injuries in the aftermath (Table 1; Figure 2).

## 3. Before the Event: Prediction of Athlete’s Injury Risk

In the studies included, both conventional statistical and AI-based methods were used. Similar to ML methods, statistical methods can be applied to raw data once a relationship is identified. Although some might argue linear and logistic regression are themselves ML techniques, there are important distinctions to be made between classical statistical learning and machine learning [27]. Most studies use ML or statistical models for inferences or predictions about biological systems. Statistical methods achieve this purpose by creating and fitting a project-specific probability model, which enables us to compute a quantitative measure of confidence that a discovered relationship describes a “true” effect that is unlikely to result from noise [28]. In contrast, ML focuses on using general-purpose learning algorithms to find patterns in larger and richer datasets [29]. This makes them useful to deal with “wide data” where the number of input variables is greater than the number of subjects. ML also makes fewer assumptions about the data generation systems. Compared to conventional statistics, which was initially designed for data with fewer input variables and sample sizes, ML can handle larger sample sizes [30,31]. As the number of input variables and associations between them increases, the model that describes them becomes more complicated, which blurs the boundaries between conventional statistics and ML [32]. Nevertheless, both techniques are invaluable in understanding the relationships existing within biological systems.

In professional sports, missed athletic events due to injury is expensive. Missed games due to injuries led to a revenue loss of USD 344 million during the 2014–2015 National Basketball Association season, while over USD 521 million was spent by the National Football League in the management of injured players [11,33]. AI technology has the potential to predict the risk of some injuries during the training phase. Several studies have evaluated the use of commercially available devices to collect training data among users and validated their use in the non-professional population. For example, Skazalski et al. showed that the commercially available Vert device was able to track an athletes’ progress to estimate the likelihood of injury among volleyball players during training and competition [9]. In another study, Chen et al. used a wearable heat-stroke-detection device with physical sensors to measure galvanic skin response, heart rate and body temperature for the prediction of heat strokes in exercising individuals [10]. Rodriguez et al. compared a combination of the dynamic Bayesian mixture model (DBMM), convolutional neural networks (CNN) and long short-term memory (LSTM) network to process physiological and positional data as sequential features for action recognition. They defined seven different actions, including running; running with the ball; passing; walking; walking with the ball; shooting; and jumping during football, and reported classification accuracies up to 80.54%. Karnuta et al. used six different algorithms (LR, random forest, k-nearest neighbours, naïve Bayes, XGBoost, and top 3 ensemble) to create 84 different models to predict seven clinical outcomes, namely, next-season injury, next-season knee injury, next-season back injury, next-season hand injury, next-season foot/ankle injury, next-season shoulder injury and next-season elbow injury. The models had predictive accuracies of 70% and 94.6% accuracy for baseball and hockey players, respectively, and outperformed conventional regression models in 93% of cases [12]. Although this may be due to the diversity and granularity of data processed, this advantage is not always present, and there can be instances where conventional statistical methods may be favourable. Given the burgeoning market for wearable devices for the prediction of injuries, the use of AI is a worthwhile investment in selected contexts to build risk prediction and stratification models with better reliability.

Athletes undergo high levels of mental and physical stress, especially in preparation for high-stakes sports events. AI tools may have the ability to use vital signs to identify levels of stress, anxiety and depression among sportspersons. For example, Coutts et al. recorded heart rate variability using fitness bands with biosensors and trained deep neural networks to characterize one’s mental health with up to 83% accuracy [16]. The earliest wearable devices tracked heart rate but were unable to meaningfully interpret it towards clinically relevant outcomes. Traditional methods of analysing heart rate variability have shown poor predictive performance, primarily due to their inability to handle large quantities of complex data collected. Using a long short-term memory (LTSM) network, a type of recurrent neural network (RNN) designed for time series analysis, the authors developed a reliable ML model that predicted stress level based on heart rate variability. In another study, Uematsu used a combination of an LTSM network, logistic regression and support vector machines (SVM) to forecast stress levels using 1231 overlapping 8-day sequences of data from 142 participants [17,34]. Overall, AI-based devices may be used to track athletes’ well-being and aid in optimising their performance, not just their physical health.

## 4. Before and during the Event: Optimisation of Athletic Performance

The generation of large volumes of data by wearable sensors allows for identifying patterns in athletic performance. Continuous real-time data before, during and after training as well as during the event can be analysed to modify and improve athletic performance. For example, Novatchkov et al. described the use of sensors to monitor force, displacement, velocity and duration variables during resistance training sessions, and pointed out ways to improve the technique of weight-lifters [13]. Sensors are most useful in identifying patterns in complex movements. For example, several studies have used AI algorithms to analyse hand–wrist motions, posture and stances as well as 3D positional systems to improve golfing performance [14]. As of 2011, the TaylorMade golf database has datasets for over 500,000 golf swings, all of which can be used to improve the swing technique of golfers. A similar effort has also been carried out in baseball, basketball, cricket and long-distance running [18,19,20]. For example, Li et al. describe a bespoke “artificial intelligence assistance system” to aid in-game decision making on three layers, namely, database, processing and display layers. The database layer compiles tactical data to build a data warehouse, which is used by the processing layer to output a visual display of the processed data into decisions. The authors also describe dynamic modes, which require inputting of in-game parameters to calculate the probability of various situations, and choose the best substitution, the best tactical combination and other data. Such a wearable platform provided real-time corrective feedback based on multidimensional physiological data collected from a body sensor network [21]. In doing so, sensors transform themselves into wearable coaches that direct the athlete’s movements [35].

Perhaps, the most chaotic sports environment is that of professional racing such as the Formula 1 Grand Prix, where AI can be especially useful. Racing events generate large volumes of continuous data on both drivers and vehicles. AI-based systems not only use data to inform how driving performance can be improved, but are also used to run simulations of different race decisions and strategies (pit stops, tyre changes, use of drag reduction systems). This has led to notable collaborations between racing teams and software companies, with the notable example being the Red Bull–Oracle brands [36]. Overall, both individual and team sports present unstable environments that are challenging to coach. Using sensors, we are able to understand the events better due to the large volume of data collected. Simple statistical analysis of these data that were previously unmeasured generate improvements in how these sports are played, even without the use of AI, and can solve the problem. Although AI has the potential to multiply this impact, it is important to acknowledge that AI is not an easy solution to manifest, and, furthermore, to address the fact that AI requires an immense volume of effort and resources to create, validate and apply these methods for real-life application in sports. Hence, we should better select the specific issues and problems we want to use AI to tackle and apply it in a more tailored manner.

## 5. After the Event: AI-Based Wearable Devices as Diagnostic Systems

The basis of machine learning lies in the processing of large amounts of data via algorithms that engage in various types of learning to detect or identify patterns, which is also a central tenet of diagnostics. Certainly, several studies have shown how automatised pattern recognition can be used in both numerical data and visual images to diagnose diseases. Wearable devices have been extensively used to monitor athletes and attain an assessment of their physical health. There have been notable reports of cardiovascular and respiratory conditions that have either led to the harm of athletes or prevented their participation in sports. In response, developers have created several wearable devices to monitor heart rate and blood pressure continuously to identify major aberrations that may signify a notable cardiac event, including arrythmias [22,23,24]. Studies have also shown how AI can be used to not only predict cardiovascular events but also their long-term impact such as heart failure, adding more utility to the diagnostic potential of wearable devices [25].

## 6. After the Event: An Opportunity to Improve Patient Experience

Globally, patient experience has grown increasingly important in the quest for providing patient-centred care. For example, the publication of the Next Stage Review by Lord Darzi has emphasised the importance of patient-reported outcome measures (PROMs) to evaluate the quality of healthcare service provision in the UK [37,38]. In the post-injury phase, athletes often undergo a period of rehabilitation, physiotherapy and treatment where wearable technology can be of use. In one study, Bloomfield et al. used machine learning to analyse data from wearable devices post total knee arthroplasty [15]. The various metrics obtained from the devices were modelled to correlate higher PROMS in the cohort that also had better post-operative functional clinical outcomes. In later work, Nwachukwu et al. developed an algorithm based on the LASSO regression technique and trained the model on 898 patients with femoroacetabular impingement [26]. The AI model was able to show that factors such as mood disorders, the prolonged duration of symptoms and high preoperative outcome scores were predictive of PROMS specific to hip surgery. Through this, AI-based wearable technology has an important role in the peri-operative rehabilitation of patients undergoing specific procedures. Not only do AI systems predict post-operative course, but they are also able to correlate them with the minimally clinically important difference (MCID) [39]. MCID refers to the minimum change in PROM scores that patients perceive as beneficial or clinically meaningful. Identifying patients who are at risk of not achieving a PROM-related MCID in the pre- and post-operative phases can be important in allocating more resources to monitor this cohort of patients and provide them with more support. In this regard, AI can improve clinical decision making and patient care by informing presurgical discussions of likely outcomes.

## 7. Challenges and Areas of Future Work

There are many challenges that face the incorporation of AI into wearable devices [40]. Firstly, obtaining high-quality data is difficult with wearable technology given the variations in spatial temporal and data resolution, which becomes more complicated when one or more devices have to be unified to collect multiple data types to generate a full picture of the body. There is also an inherent bias towards collecting data from only those who can afford these sensors, which creates a socio-economic bias in the models [41]. Other challenges such as missing data, outliers, signal noise and artifacts can introduce large variations and produce erroneous algorithms. For example, sensors that monitor heart rate also have to discern artefacts created by arm motion during physical activity. More complex sensors that can collect and transmit cleaner data need to be developed to overcome this. Even if high-quality data are collected, the transmission of the data from wearable technology to processing platforms is both time and resource intensive. Perhaps, the biggest and most difficult challenge to tackle is ensuring that the data are handled with the highest level of security. Furthermore, wearable devices are privy to an existing problem within most healthcare systems, where different healthcare databases are not connected. When patients use different devices to capture different types of data, the complexity of the data generated can be better managed if device-to-device communication between wearable devices with different computational power is achieved.

The integration of AI technology and wearable devices into healthcare systems requires us to define their role, their capabilities and limits, all of which will require more input from various stakeholders, including hospitals, patients, programmers, policy makers, insurance companies and companies that produce wearables. Perhaps the biggest challenge to their adoption will be patient acceptability. About 50% of consumers who buy a wearable stop using it, and this happens within six months in approximately a third of them [42,43]. One study showed that just 50% of patients felt the use of AI within wearable technology was an important opportunity, and 11% even noted it as a harm [44]. The surveyed patients were concerned that technology could exploit and misuse their data and dampen the human element aspect of healthcare. More efforts to educate the public on how AI helps physicians, their abilities and restrictions are necessary to increase patient acceptability and user adoption.

## 8. Conclusions

Artificial intelligence is a promising avenue for integration into wearable technology. While the use of AI brings with it a separate set of challenges, it also has numerous advantages in its ability to translate the use of wearable technology on a large scale. AI can improve the way injury prediction models work; increase the diagnostic accuracy of risk stratification systems; provide a reliable method for the continuous monitoring of patient health data; and enhance the quality of the patient’s experience. However, at this point, the technology and logistics underlying wearable devices themselves are still at early stages and need further work. Further work is necessary from physicians, programmers, policy makers and makers of wearable devices to overcome the logistical and practical barriers, and to ensure that wearable devices are smoothly incorporated into the digital platforms of healthcare systems.

## Figures and Tables

**Table 1:** Studies reporting sensors’ use in the different phases of sports and the outcome measures.

Author	Year	Country	Design	Sample Size	Sex (% of Males)	Age	Sport/Activity	Outcome Measures	Sensor	Conclusion
Skazalski et al. [9]	2018	Qatar	Comparing IMU data with visual observation of jumps	14	100	Not Specified	Volleyball	1. Jump Count recorded by IMU device, compared with visual observation 2. Jump height recorded by IMU device and compared with visual observation	Vert Classic (Model #JEM) with Vert Coach Application (version 2.0.6)	Vert device demonstrates excellent accuracy counting volleyball-specific jumps. Vert Device can be used to monitor athlete jump intensity
Chen et al. [10]	2018	Taiwan	Using a Wearable heat-stroke-detection device (WHDD) to monitor a runner’s physiological information	1	100	35	Running	1. Galvanic skin response. 2. Heart rate. 3. Body temperature. 4. Ambient temperature. 5. Ambient humidity. 6. Predicted risk of heat stroke. Above measures all recorded during a predertemined running programme	Custom WHDD with GSR, MLX90614 and SHT75 sensors.	WHDD detected the trend in a runner’s physiologcal information in advance of exercise intensity. The WHDD could specifically prevent the occurrence of heat stroke.
Lewis [11]	2018	USA	Cross sectional study	627	100	Not specified	Basketball	1. Injury events. 2. Player fatigue. 3. Performance load (total rebounds and field goal attempts).	Random-effects, multi-level logistic regression model	Higher levels of fatigue and workload led to greater injury risk. With these constant factors, a higher injury risk was associated with greater NBA experience and below average height.
Karnuta et al. [12]	2020	USA	Descriptive Epidemiology Study	139,783	100	Not Specified	Baseball	Predictions for future injury risk based on logistic regression and machine learning algorithms.	Logistic regression, random forest, k-nearest neighbours, Naïve Bayers, XGBoost, Top 3 Ensemble. Models were built usnig scikit-learn Python library (Version 0.20.3) and XGBoost (Version 1.0.2)	Advanced machine learning models outperformed logistic regression and demonstrated fair capability of predicting whether a publicy reportable injury was likely to occur.
Novatchkov and Baca [13]	2013	Austria	Descriptive Study	15	53	24.6	Weight Training	Force displacement parameters measured from a weght leg press machine	Weight leg press machine equipped wht a load cell (PW10A or PW12C3, Hottinger Baldwin) and a rotary encoder (DP18, Altmann). Modelling of signals by multilayer pattern recognition networks based on the Levenberg-Marquardt algorthm.	Computer based feedback frameworks can be used for analysis of performance during workouts.
Ghasemzadeh et al. [14]	2009	USA	Quantitative analysis of golf swings using BSN	4	75	20–35	Golf	Degrees of wrist rotation during segments of a golf swing	TelosB from Xbow	Body Sensor Networks can provide information on the quality of a golf swing with respect to the angle of the wrist rotation
Bloomfield et al. [15]	2019	Canada	Cross sectional study	68	34	65.6 ± 9.1	Timed-up-and-go tests	Post-operative recovery	custom wearable system	Wearable sensors during instrument functional tests during clinical visits and using machine learning to parse complex patterns can reveal clinically relevant parameters
Coutts et al. [16]	2020	UK	Prospective cohort study	100 in trial 1; 799 Iin trial 2	38; 224	18–38; 18–69	Cycling	Heart rate; Perceived Stress Scale; Depression Anxiety Stress Scale; State and Trait Anxiety	Biobeam band; Deep Neural Networks (LSTMs)	Classification accuracy of up to 85% with the current AI model and biosensor.

## Data Availability

All data was extracted directly from included studies.
